# Urban Road Surface Discrimination by Tire-Road Noise Analysis and Data Clustering

**DOI:** 10.3390/s22249686

**Published:** 2022-12-10

**Authors:** Carlos Ramos-Romero, César Asensio, Ricardo Moreno, Guillermo de Arcas

**Affiliations:** 1 Acoustics Research Centre, University of Salford, Manchester M5 4WT, UK; 2 ETSI Sistemas de Telecomunicación, Departamento de Ingeniería Audiovisual y Comunicaciones, Grupo de Investigación en Instrumentación y Acústica Aplicada (I2A2), Universidad Politécnica de Madrid, 28031 Madrid, Spain; 3 Institute for Chemical-Physical Processes of the Italian Research Council (CNR-IPCF), Via Giuseppe Moruzzi 1, 56124 Pisa, Italy

**Keywords:** road surface, pavement condition, tire-road noise, unsupervised machine learning, data clustering

## Abstract

The surface condition of roadways has direct consequences on a wide range of processes related to the transportation technology, quality of road facilities, road safety, and traffic noise emissions. Methods developed for detection of road surface condition are crucial for maintenance and rehabilitation plans, also relevant for driving environment detection for autonomous transportation systems and e-mobility solutions. In this paper, the clustering of the tire-road noise emission features is proposed to detect the condition of the wheel tracks regions during naturalistic driving events. This acoustic-based methodology was applied in urban areas under nonstop real-life traffic conditions. Using the proposed method, it was possible to identify at least two groups of surface status on the inspected routes over the wheel-path interaction zone. The detection rate on urban zone reaches 75% for renewed lanes and 72% for distressed lanes.

## 1. Introduction

Due to continuous improvements in technologies applied to transportation systems, from autonomous driving to e-mobility, it is more common to find sensors embedded in vehicles that allow continuous scanning of the road environment. One of the crucial elements of this environment is the superficial pavement condition which is closely related to traffic safety and rolling noise emissions [1,2].

The Tire-Pavement Interaction Noise (TPIN) is a complex phenomenon which depends on a number of parameters, such as tire characteristics (e.g., tread pattern, inflation), driver influence (e.g., speed, acceleration), environmental conditions, surface contamination (e.g., wet, dry, dusty), and the parameter on which the present work will focus, the superficial asphalt condition [3,4,5,6].

Even though noise reduction properties of low-noise pavements decrease over time, resulting in heterogeneous tire-road noise generation along the route [7], the road inspection activities are mainly focused on roughness, skid resistance, and distress regarding functional and asphalt serviceability [8]. These conditions of the rolling surface are closely related to the noise generation from the wheel-path interaction zone, including both the megatexture and the macrotexture [9,10].

Traditionally, the identification and evaluation of asphalt defects has been performed by labour-consuming visual inspections. These activities have some subjective assessments due to experience and the assessor’s judgment of the catalogued defects. Other approaches, such as coring tests, are also available as mechanical techniques [11]. These methods are time-consuming, require further laboratory analysis, and are not easy to apply to large road sections.

Conversely, new remote sensing methodologies can address some of these shortcomings; for example, analysing large road sections is possible while handling large amounts of data. These methods include tools such as ground penetration radar, infrared thermography [12], laser scanning [13], image-based [14,15], vibration-based [16,17,18], and acoustic-based [19] methods. As these techniques are not mutually exclusive, more than one technique can be used simultaneously [10,20].

In particular, the acoustic-based methodology exploits the information from the rolling noise measurements, (i.e., Tire Pavement Interaction Noise (TPIN) [21], which is dominant over powertrain noise above the “crossover speed”, usually 35 km/h for combustion engine cars and lower speeds for electric vehicles [22].

The objective of this paper is to present an acoustic-based approach for unattended discrimination of the changes of the road surface conditions over the wheel-track path through the analysis of the tire-road noise and unsupervised machine learning (UL). Thus, the resulting clusters are presented accordingly the road sections of similar asphalt status. Although the obtained noise measurement data would also gather information about other aspects present during rolling, such as acoustical comfort inside the car, tire-pavement adhesion mechanism, vibrations, and aerodynamic phenomena, their identification is out of the scope of the present work. This study addresses the influences of driving conditions into the analysis, such as speed and acceleration of the vehicle, and several types and degrees of damage could be found on a typical route. However, the design of a dataset with all distress types for supervised classification could be a difficult and time-consuming task. Hence, UL algorithms will be included in superficial pavement monitoring of three road circuits. Table 1 shows a summary of the state-of-the-art research focused on road materiality studies based on the acoustic information contained in TPIN signals.

Although some standards for rolling noise measurement in near field have been implemented, such as CPX and OBSI, the data analysis from several sensors needed for their configuration can be time and computationally consuming tasks for other purposes added to policy noise level reports. Behind-The-Tire (BTT) measurement setup is a practical option, which allows to analyse the relative influence of pavement texture on tire-road noise through a simpler electroacoustic setup configuration [21,23]. The BTT technique has provided good performance in data acquisition stage for further identification of the actual condition of roads [5,24,25,26].

**Table 1:** Literature review: Road condition identification based on vibroacoustic data.

Author	Sensors		Method	Classification						Feature Extraction *	Learning Approach		
	Sound	Vibration		Roughness	Asphalt Type	Wet/dry	Anomalies	Macro-mega Texture	Road Labelling		Classifier Algorithm *	Supervised	Unsupervised
Masino et al. [27]	x		Tire cavity sound measurement		x					PSD	SVM, ANN	x	
Ambrosini et al. [28]	x		Multichannel array	x						MFCC	CNN	x	
Doğan [29]	x		Behind the tire (BTT)		x					MFCC, PSC, LPC	ANN	x	
Paulo et al. [30]	x		Close-Proximity method (CPX)		x					1/3 OCT	Bayesian	x	
Kongrattanaprasert et al. [31]	x		Coast-by method			x				Power spectrum	ANN	x	
Alonso et al. [5]	x		BTT			x				1/3 OCT	SVM	x	
Abdic et al. [32]	x		BTT			x				Mel-Frequency scale.	SVM, RNN-LSTM	x	
Pepe et al. [33]	x		Internal and external microphones			x				MFCC	CNN	x	
Kalliris et al. [25]	x		On board single microphone			x				1/1 OCT	SVM	x	
Gueta and Sato [17]		x	On board smartphone monitoring		x		x			Peaks on the signal. WT	SVM, LDA, KNN	x	
Ramos-Romero et al. [26]	x		BTT		x		x			1/3 OCT	KNN.	x	
Safont et al. [34]	x	x	10 channel sensor system		x					256 features	PCA, LDA, SVM, RFC	x	
David et al. [35]	x		Microphone pointed to the wheel	x	x					1/3 OCT—speed	Clustering		x
Ganji et al. [36]	x		CPX					x		MFCC	SVM	x	
Zhang et al. [37]	x		BTT					x		PCA	Statistic model	x	
Van Hauwermeiren et al. [38]	x	x	Opportunistic sensing box						x	Sound levels and acceleration. DAE	DAE	x	
Del Pizzo et al. [9]	x		Tyre Cavity Microphone		x			x		1/3 OCT	Statistic model	x	

* Power spectral density (PSD). Power spectrum coefficients (PSC). Mel-frequency cepstrum coefficients (MFCC). Linear predictive coefficients (LPC). Principal components analysis (PCA). Wavelet transform (WT). Octave frequency spectrum (OCT). Support vector machine (SVM). Linear discriminant analysis (LDA). Convolutional neural networks (CNN). Artificial neural networks (ANN). Denoising autoencoder (DAE). Recurrent neural networks (RNN). Long-short term memory (LSTM). K-nearest neighbour (KNN). Principal component analysis (PCA). Random forest classifier (RFC).

Most of the literature in this field, exploiting the advantages of supervised machine learning in surface-asphalt detection using the frequency features of TPIN measurements, reports high accuracy on classification tasks, for instance the surface-road type, materiality class or wet-dry condition. The supervised method has been effective for these tasks, although it requires a labelled database with enough examples of all possible deterioration classes to identify them on the actual route. However, the road upper layer to be inspected might have many forms and degrees of deterioration, and several of them can even be unknown; especially, when the inspection is over long distances. In addition, the difficulty of collecting this complete database is the reason for the decision to use the unsupervised approach for monitoring the superficial asphalt condition along the wheel-path interaction zone.

This paper is organized as follows: Section 2 presents a detailed structure of the methodology, including data acquisition, signal processing, the UL algorithm, and geoprocessing. Then, in Section 3, the obtained results applying the methodology on urban roads are presented. Section 4 discusses the experimental results, the benefits of the application of this experimental approach, and the detected limitations. Finally, main conclusions of this work are detailed in Section 5.

## 2. Materials and Methods

**Figure 1:**
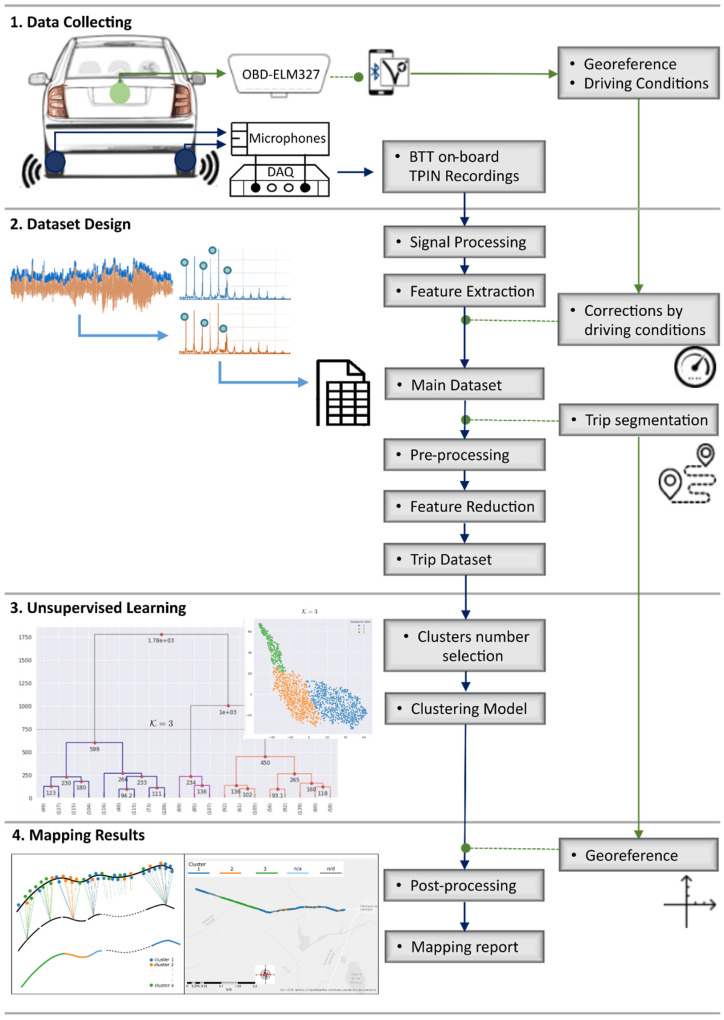
Methodological flowchart.

The implemented method consists of four main steps, as depicted in Figure 1.

Firstly, the data are collected from three different types of input, such as the recordings of the TPIN signal, speed, and acceleration from the vehicle’s electronic control unit via the On-Board Diagnostics (OBD) port, and coordinates of the surveyed routes from a smartphone GPS. Afterwards, the dataset construction is performed through sound signal processing by frequency domain transformations, feature extraction, and driving condition dependences. The conformed dataset can be sliced by geographic areas or specific streets to deepen distress road identification.

Next, the similarities between the acoustic footprint data are clustered using UL algorithms. Finally, the available data from GPS were used to visually represent the detected differences in road conditions. Since the GPS of the mobile phone has an accuracy of 4.9 m, the geolocation data is not intended to determine the location of a specific type of deterioration, but rather a generalized inspection of the road surface.

### 2.1. Data Collecting

**Figure 2:**
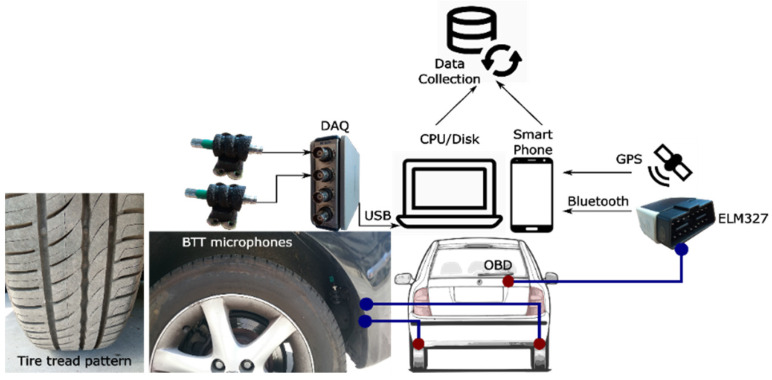
Sensor’s set-up.

As far as possible during the data recording rounds, some constraints of the experiment were conserved to reduce variability factors and facilitate the subsequent interpretation of results. Only one class of tire tread pattern was installed on the experimental vehicle, which is depicted in Figure 2. This tread pattern is recommended for urban mobility in all seasons by the manufacturer (Pirelli-Cinturato P1™ Verde). The tire inflation pressure was checked before each experiment and kept constant at 2.2 bar. A single driver drove the routes to minimize variability in driving behaviour. In addition, the own vehicle’s mass was checked to ensure that it remained the same as much as possible. For this purpose, only the driver, regulatory equipment and data acquisition hardware were carried in the vehicle during the experiments. In addition, the measurement runs were driven with the fuel tank above its half-capacity whenever possible.

The TPIN data acquisition campaign was carried out during fall season under stable weather conditions, i.e., average air temperature maintained between 22–25 °C and humidity of 40–50%. Only dry asphalt was considered in the experiments.

Sound pressure signals were collected by using a unique instrumented diesel passenger car, equipped with two 1/2” type-1 microphones and BSWA-MA231 preamplifiers, according to the BTT technique. Although there is not an international standard for this simple instrumentation technique, it offers certain advantages for inspections. For applications in opportunistic scenarios, the sensor configuration is performed with the minimum disturbance to the vehicle structure, no additional trailer is required, and the microphones remain hidden during inspections.

The microphones were connected to an NI-9234 Data Acquisition (DAQ) device with sample rate of 51,200 Hz. The DAQ software is controlled by a portable PC on board. Although the measurement tasks were performed with two microphones, one on each rear wheel, the data captured by each microphone were processed independently. This means that the instrumentation setup simulates two vehicles of the same type making the same run with only one BTT microphone.

Simultaneously, an ELM327 interface connected at the car OBD port sends the driving parameter data to a smartphone via Bluetooth at 1 Hz. Because the PC was connected to the mobile network through the Wi-Fi smartphone portable hotspot, their clocks were matched to enable recordings using a single clock.

### 2.2. Dataset Design

The method seeks to recognize the differences between the sounds coming from different road conditions when the tire rolls over them. In this context, the information that makes the sound comparison between the signals is extracted from the TPIN registers and then constitutes a data set. The selected features are then expected to contain the relevant information of the input data.

The first step is to pass the audio data through a cut-off high-pass filter from 20 Hz, which removes the unrelated signal to rolling phenomenon at lower frequencies, such as sources of mechanical vibration and nonaudible acoustical data. Then, the audio data were split into 1-s nonoverlapped frames. This frame size allows matching between the OBD and GPS data sampled at 1 Hz with the corresponding TPIN frame trough date-time data. A Hamming window was applied to reduce the discontinuity effects at the boundaries of each chunk before the transformation in the frequency domain [39]. Each audio frame is processed by the Discrete Fourier Transform (DFT). The power amplitude is computed as the absolute value from the first half of the coefficients of DFT. Subsequently, the data conformed by DFT spectrum are further processed in the filter bank.

In general, literature reports that the sound radiation of TPIN mechanisms is prominent below 4000 Hz, and it is closed related with parameters of both the pavement (e.g., wavelength texture based on the aggregate particles in the mixture, the road condition and temperature) and the wheel (e.g., speed, torque, load, and inflation) [3,40]. Moreover, the rolling noise amplitude, at frequencies below 1000 Hz, increases with texture amplitude within the texture wavelength range of 10–500 mm. Indeed, above 1000 Hz, the noise amplitude decreases with texture amplitude within the texture wavelength range of 0.5–10 mm. [3]. Additionally, the changes in asphalt macrotexture are closely related to sound levels in the frequency range between 40 and 400 Hz at a specific speed [41]. Likewise, the sound intensity level is correlated with pavement surface texture at different frequency bands, from 315 to 2500 Hz [42].

Although TPIN noise characterisation is most commonly performed in the frequency domain through the 1/n-octave bands [5,25,26,29,35], there are alternatives for noise representation that are capable of handling the subjective impression of frequency, such as Mel’s triangular filter bank.

Indeed, the triangular filter bank was typically selected for audio processing in tasks related to the non-linear perception of sound by humans [39,43]. The reason for this type of filtering approach is that passengers inside the vehicle can perceive changes in the road surface by both auditory and vibrational stimuli, so this bank filtering is generally associated with acoustic comfort in the vehicle cabin [44].

In accordance with the frequency behaviour of the TPIN described above, the triangular filter bank was designed to extract the data at frequencies below 4000 Hz.

**Figure 3:**
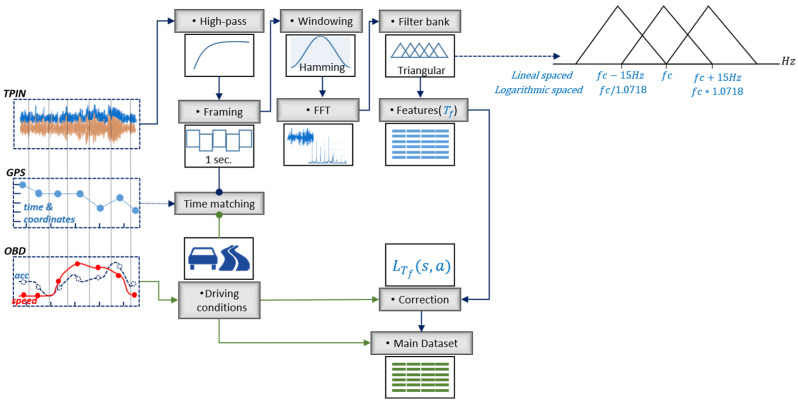
Dataset design.

On the whole, each signal frame passes through a set of triangular filters ($T_{f}=50$). The first 15 central frequencies ($f_{c}$) are linearly spaced by 15 Hz ($f_{c}$ = 65 Hz to $f_{c}=$ 260 Hz), and the next 35 central frequencies ($f_{c}$ = 278.5 Hz to $f_{c}=$ 3089.4 Hz) are logarithmic spaced [45]. Figure 3 depicts the triangular filter bank representation, and the frequency bands are described in the Appendix A.

The OBD data allow the selection of only those signal frames that were recorded at more than 35 km/h, i.e., the data that continue the process are above the crossover speed. The detailed length of the studied roadways and corresponding observations [n] are listed in Table 2.

#### 2.2.1. Corrections by Driving Conditions

To minimize the influences of driving conditions (speed and acceleration) on the frequency extracted sound features, a linear multivariate model was implemented. The data for the model were recorded under a wide speed range (35 to 60 km/h) on urban roads with the same vehicle, set of tires, and driver in order to minimize the data variability (see Table 2). The data used to derive the linear model was excluded from the data sets detailed in the results.

The speed s [km/h] and acceleration a [m/s^2^] of the vehicle registered during the trips are considered for corrections of noise level on each frequency band [35]. It is important to note that if speed and acceleration are taken as independent features, the clustering algorithm could return an incorrect detection of asphalt zones because it could group the zones by driving performance or roads constraints rather than by rolling noise and pavement conditions.

Consequently, every triangular band with sound pressure level in dB of a 1-s element [n] $L_{T{\left[n\right]}_{f}}$ on the dataset is adjusted by s and a influence by the Equation (1), where ${L^{\prime }}_{T\left[n\right]\left(f\right)}$ is the corrected level and $Bs_{T_{f}}$ and $Ba_{T_{f}}$ are the coefficients for linear regression for speed and acceleration, respectively, at the reference speed $s_{ref}=70\;\mathrm{km}/h$ [35]. Although this $s_{ref}$ value was applied to tire-road noise experiments related to road roughness [35] other values could also be considered [46].

(1) $${L^{\prime }}_{T\left[n\right]\left(f\right)}=L_{T{\left[n\right]}_{f}}-Bs_{T_{f}}{log}_{10}\frac{s\left[n\right]}{s_{ref}}-Ba_{T_{f}}a\left[n\right]$$

Then, the feature space is made up of frequency bands with the coefficient of determination$R^{2}\geq 75\%$ and $p_{value}\leq 0.005$. The bands with $R^{2}<75\%$ or $p_{-value}>0.005$ are rejected. Hence, only the bands from $f_{c}=392.8\;\mathrm{Hz}$ to $f_{c}=3089.4\;\mathrm{Hz}$ will be kept for the following stages of the experiment. These resulting 31 bands correspond to the range of useful frequencies indicated in the literature for tire/noise and pavement condition studies [42]. The selected triangular bands and the coefficients for linear regression are presented in Appendix A.

Furthermore, deterioration in certain areas of the pavement has been observed due to several punctual defects distributed over the surface. This surface irregularities generate impact noise events. To represent these noise dynamics during rolling, three additional overall-level features have been included for each observation [n] of the data. These are the peak level $L_{Peak\left[n\right]}$, equivalent continuous level $L_{eq\left[n\right]}$, and the difference between them $CF_{\left[n\right]}=L_{Peak\left[n\right]}-L_{eq\left[n\right]}$ as crest factor. Finally, the main dataset is constructed with the featured TPIN by each element [n] as $x=\left\{{L^{\prime }}_{T\left[n\right]\left(f\right)},L_{Peak\left[n\right]},L_{eq\left[n\right]},CF_{\left[n\right]}\right\}:x\in {\mathbb{R}}^{34}$.

#### 2.2.2. Trip Segmentation

Once completed, the dataset can be filtered by geographic regions for further local and specific analysis. The available GPS data tracking makes it possible to select the amount of data for the next tasks by a geospatial query. Each zone has a very specific pavement structural capacity and corresponds to the same administration. The similarity in the initial asphalt mixture type within each geographical section has been assumed. This consideration would facilitate further analysis since the variability of the noise signal can reach 10 dB due to differences in pavement texture [7]. Therefore, data segmentation allows the selection of roads for a more specific analysis purpose, e.g., roads with similar usage and traffic density.

#### 2.2.3. Pre-Processing and Feature Space Reduction

After data segmentation, the available data were pre-processed to ensure that each feature x contributed equally to the estimation of the parameters of the unsupervised model. Thus, the standardized feature $x_{std}$ was computed by Equation (2), with mean $\mu_{x}$ and standard deviation $\sigma_{x}$. These new scaled features are centered with mean 0 and standard deviation 1 [47].

(2) $$x_{std}=\frac{x-\mu_{x}}{\sigma_{x}}$$

Next, the transformation of high-dimensional data into a meaningful representation of reduced dimensionality is included through dimensionality reduction, also called the feature reduction task. As a result, feature reduction facilitates and improves the cluster discrimination, classification, visualization and compression of high-dimensional data [48].

In this regard, unsupervised nonlinear dimensionality reduction was applied by the t-Distributed Stochastic Neighbor Embedding algorithm (t-SNE) introduced by Maaten and Hinton [49]. t-SNE attempts to maintain the local neighbourhood structure of input data points $X=\left\{x_{1},x_{2},\ldots ,x_{n}\right\}\subset {\mathbb{R}}^{d}$ in low-dimensional space $Y=\left\{y_{1},y_{2},\ldots ,y_{n}\right\}\subset {\mathbb{R}}^{s}$, where s≪d, usually s=2 or 3.

The above algorithm allows us to preserve the local structure of the data through pairwise similarity based on the Euclidean distance, while preserving much of the global structure of the data [49,50,51]. This distance-based feature reduction approach has provided better results for clustering tasks than other feature reduction methods, such as principal component analysis. Although this method was suggested for the graphical representation of features in a reduced dimensional space, it can also be employed with the clustering process, as it has been recently applied in fault detections and monitoring experiments [52]. Finally, the dataset reduced space results in $y\in {\mathbb{R}}^{2}$, by t-SNE components equal to 2.

### 2.3. Unsupervised Learning: Cluster Model and Validity

Because there are no a priori labelled classes about the current condition of the inspected streets, UL may be able to explore the similarity and separability criteria among the observations in the data set. Then, two types of clustering techniques with different approaches were applied, but similar results were obtained: hierarchical clustering and probabilistic clustering. In many cases, the first attempt at grouping data set results in a clustering that may not be the most effective, so multiple clustering configurations must be studied [53].

The hierarchical approach does not apply a random initialization. Particularly, the data pooling algorithm starts with each observation as a cluster itself. These smaller clusters merge into larger ones by a series of successive fusions of the observations by minimizing the distance between clusters criteria [54]. The result of this agglomerative algorithm is a tree of clusters with distance relations. The number of clusters k or disjointed groups of data is obtained by cutting trees or dendrograms at a desired level [55].

Alternatively, the probabilistic Gaussian Mixture Models (GMMs) technique form ellipsoidal-shaped clusters based on the iterative expectation-maximization algorithm. It provides the basis for the Bayesian Gaussian Mixture Model (BGMM) [47,55,56].

The approximation of the minimum number of clusters could be established by the “elbow diagram”. The elbow method helps to determine the optimal number of clusters K by means of the sum of squared errors or inertia (SSE) function minimization [47]. The inflexion point “elbow” of the SSE vs. K plot shows the optimal number of clusters.

**Figure 4:**
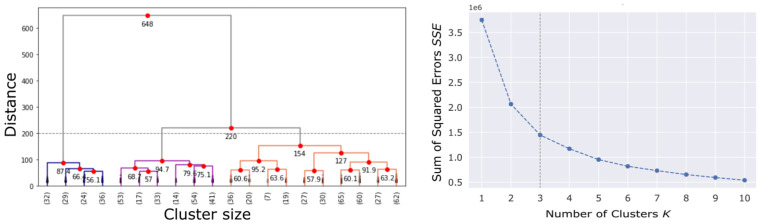
Selection of number of clusters k=3 by Dendrogram (**left**) and Elbow plot (**right**).

An example of the selection of the number of clusters k=3 by both described clustering methods is depicted in Figure 4. Clustering algorithm results in a unique cluster label $C_{k}:k\in \left\{1,2,\ldots ,K\right\}$ for each element of the dataset. This label is derived from the order in which the clusters have emerged.

### 2.4. Geo-Procesing of Results

The representation of all the passes over the inspected route is carried out by a basic geo-processing stage. In this process, the cluster type assigned to each element of the dataset was plotted on a map by GPS tracking data. However, after the cluster assignment task, the elements per cluster class might appear during normal driving with a certain level of randomness because the area of the asphalt on which the wheel contacts is not always the same. Moreover, defects may not be reached by the wheel in a single pass, especially in deteriorated areas with very local defects; in that case, the deteriorated area of the road upper layer is not registered just because the defect has not been passed over. Therefore, the possibility of detecting defects increases with the number of records belonging to the same street segment.

To address this drawback, the travelled road is first segmented, and then each element of the clustered dataset is related to the nearest piece of the road line. Road segments were set every 20 m for urban roads. This segment length allows us to include enough cluster elements in each segment when velocities are not constant. Finally, the segment label is assigned by the mode Mo of the cluster type events $C_{k}{|}_{segment}$ associated with it, according to Equation (3). This information reveals a continuous report of the asphalt status along the inspected route. The basic geo-processing procedure was also applied in a previous work [26].

(3) $$Label_{segment}=Mo\left\{C_{k}{|}_{segment}\right\}$$

When a segment of route is classified as multimodal, the label legend *not assigned* will be present in the mapping report as a “n/a”. In the same way, the segments without available data (due to stop-car or low-speed events) will be present as “n/d”, from legend *no data*.

## 3. Results

The performance of the introduced acoustic-based methodology for surveying road condition was tested on urban roads (see Table 2).

### 3.1. Reference Route

**Figure 5:**
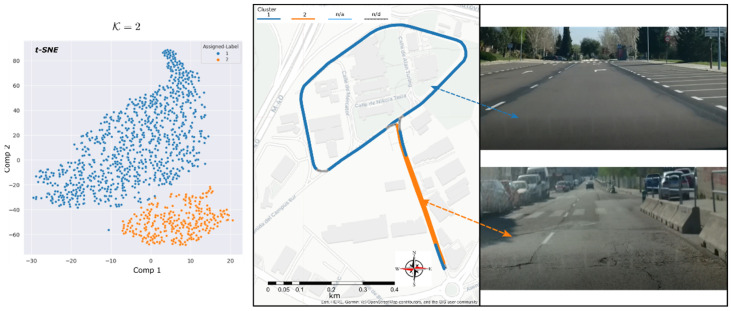
Reference-route data clustering results. t-SNE clusters formed (**left**). Geopositioned clusters (**middle**-**right**).

The first experiment was carried out in the Reference-route. The benefit of applying the proposed methodology on this route is to evaluate the clustering performance on a controlled and known road environment. The road has two different types of superficial conditions (Figure 5) which includes the last renewed pavement (6 months before the data acquisition) conforming the main ring-shaped route, and the straight access to the ring where the distress sections are presented, such as longitudinal, transversal and alligator cracking.

**Figure 6:**
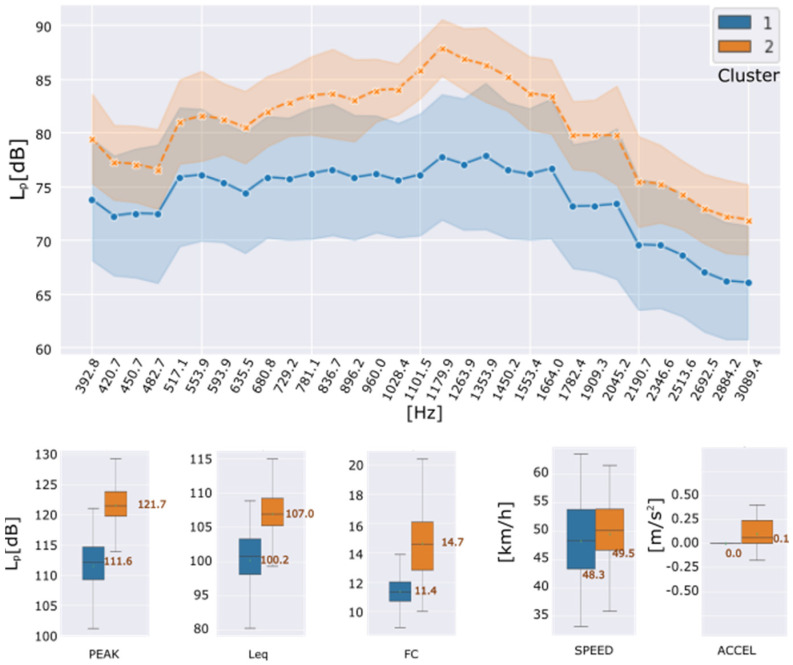
Reference-route data clustering results. Features and driving conditions by formed clusters: Frequency domain (**up**); Peak level, Equivalent continuous sound level, Crest Factor, Speed and Acceleration (**down**).

For the feature reduction task, the t-SNE algorithm was fitted to respond closer to the known condition of the reference surface road, i.e., t-SNE components (n = 2) and (perplexity = 30). This last one is a parameter that means (loosely) how to balance attention between local and global aspects of the data [57]. Figure 5 shows the correspondence between the feature reduction step and the resulting clusters for pavement-condition zones. Figure 6 shows the clustered original features for the two resulting types of asphalt conditions.

Frequency features are showed in Figure 6 as the mean spectrum of the dataset for each cluster with its standard deviation. Temporal features are shown as box and whisker plots.

The cluster $C_{2}$ has the highest readings of noise levels per band, mainly between 896.0 Hz and 1553.4 Hz bands. Since the initial materiality of the route is considered the same, and the driving speed and acceleration are quiet constant into the selected data it can be assumed that the section assigned to cluster $C_{2}$ would correspond to the most deteriorated surface condition. Likewise, the differences in the means of the global noise level indicators between clusters $C_{2}$ and $C_{1}$: $\Delta L_{Peak\;}\approx 10\;\mathrm{dB}$, $\Delta L_{rms\;}\approx 7\mathrm{dB}$ and $\Delta CF_{\;}\approx 4\;\mathrm{dB}$; reinforce the premise that the route sections with distressed conditions is located on the route assigned to cluster $C_{2}$.

Subsequently, the geographic processing of the instances assigned to each cluster was carried out through the stage described in Section 2.4. It was possible to visualise the sectors of the surveyed route according to the similar behaviour of the rolling noise footprint obtained from the clustering stage in Figure 5.

### 3.2. Urban Avenue

**Figure 7:**
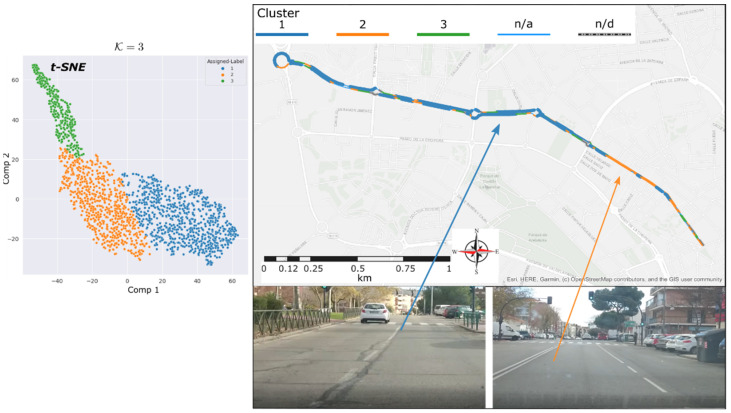
Urban avenue data clustering results. t-SNE clusters formed (**left**). Geopositioned clusters (**right**).

The second group of data refers to a single avenue in urban environment. It was known that maintenance work had been done previously, and a short section of this road had been completely repaved. Thus, the present experiment was motivated by the evaluation of the model’s ability to identify both distressed and renewed areas. For this experiment, the resulting number of clusters was k=3, as is depicted in Figure 7.

**Figure 8:**
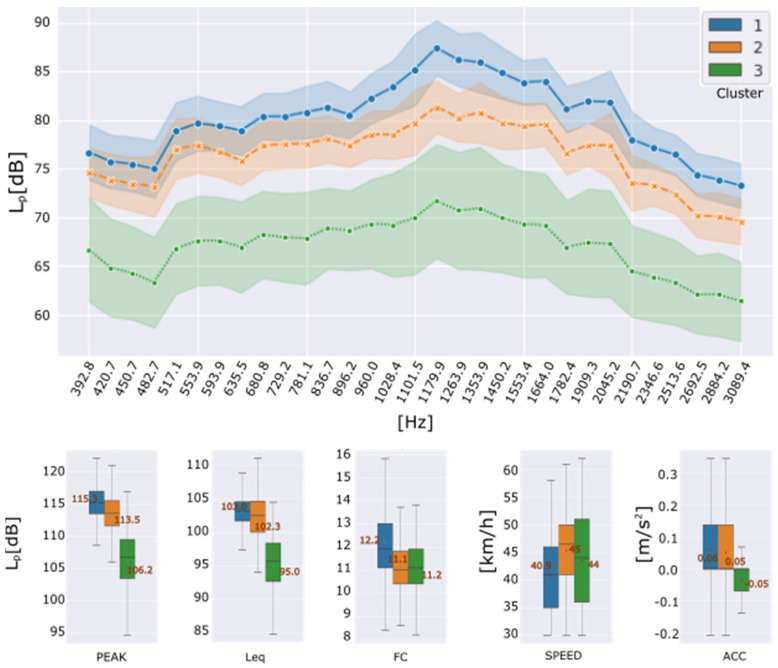
Urban avenue data clustering results. Features and driving conditions by formed clusters: Frequency domain (**up**); Peak level, Equivalent continuous sound level, Crest Factor, Speed and Acceleration (**down**).

Subsequently, the instances assigned to each cluster could be displayed in their original domain, i.e., according to noise levels by frequency bands and by overall noise levels. Although the clustered noise spectrum does not show more than 3 dB difference between the average levels of each cluster (Figure 8) and the main values of the $L_{Peak}$ and $L_{eq}$ values of $C_{1}$ and $C_{2}$ are closer to each other <3 dB, the $C_{3}$ is 6 dB lower than the other two clusters. The cluster $C_{3}$ corresponds to the data in driving deacceleration. The resulting clusters could be reported by the surveyed route line illustrated in Figure 7. In the mapped route report two main segments can be identified the one corresponding to cluster $C_{1}$ and a shorter one corresponding to cluster $C_{2}$. The third cluster appears located in the street intersections, roundabout approximation zones and pedestrian crossings which could explain the data obtained during the car’s deacceleration. Cluster $C_{3}$ combines the lowest TPIN levels on feature plots. Nevertheless, this is not related completely with the superficial condition of the wheel-path interaction zone but with the driving conditions during the experiments.

### 3.3. Urban Street Circuit

**Figure 9:**
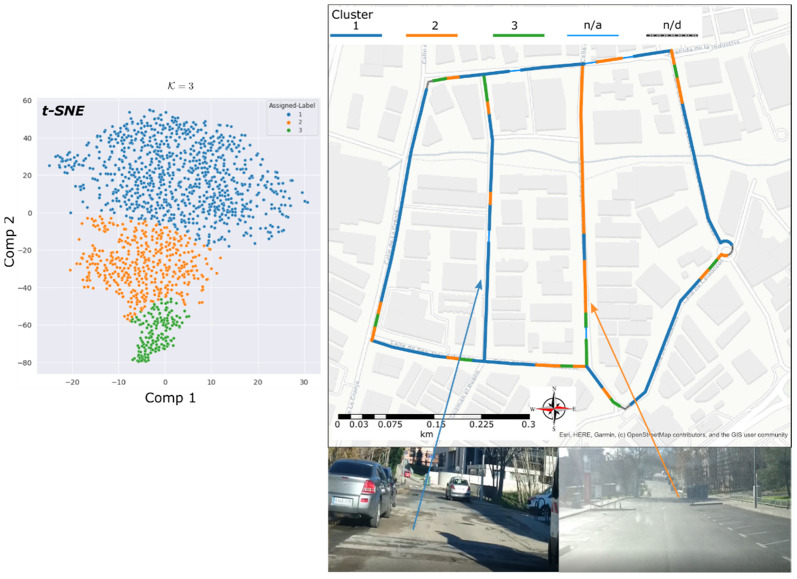
Urban Street circuit data clustering results. t-SNE clusters formed (**left**). Geopositioned clusters (**right**).

The reason of this last experiment was to test the identification of the condition of the wheel-path interaction zone among several neighbouring streets. These streets, with similar characteristics, such as vehicle density flow and travel speed, do not necessarily have the same asphalt condition, but in general, the initial installed materiality could. Figure 9 shows the obtained results correspond to cluster number k=3.

**Figure 10:**
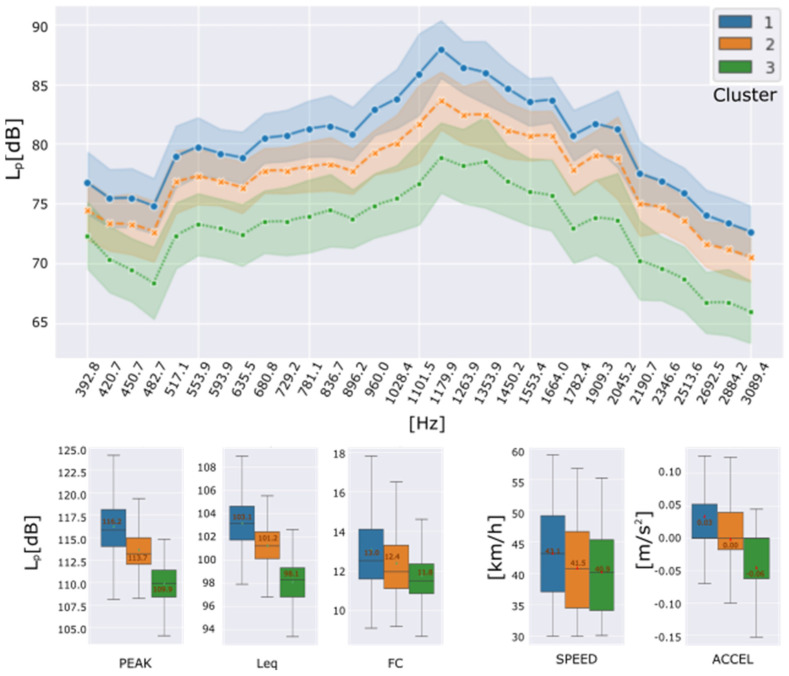
Urban Street circuit. Features and driving conditions by formed clusters: Frequency domain (**up**); Peak level, Equivalent continuous sound level, Crest Factor, Speed and Acceleration (**down**).

Figure 10 shows the averages of the characteristics in frequency and time domain, as well as their standard deviation for each cluster assigned to the studied data. It is evident that instances assigned to cluster $C_{1}$ have the highest amplitudes, followed by cluster $C_{2}$ and cluster $C_{3}$ with lower amplitudes. In general, a difference of about 5 dB is observed between the band spectrum means and consecutive clusters. This slight difference in the frequency characteristics of clusters $C_{1}$ and $C_{2}$ could indicate that the track section assigned to cluster $C_{1}$ is in worse condition than the section assigned to cluster $C_{2}$.

Besides, cluster $C_{3}$ shows the lowest amplitudes, and it could be associated with a quieter routing condition than the other clusters found. Its behaviour is like the third cluster of the previous experiment’s data set, i.e., the urban avenue. Hence, we can deduct that cluster $C_{3}$ is not related to the superficial condition of the wheel-path interaction zone.

Mapping report depicted in Figure 9, a marked zone assigned to cluster $C_{2}$ is dominant in almost a whole street of the inspected group. Because of the features on cluster $C_{2}$ are lower than $C_{1}$, a better quality of asphalt is assumed. The greater section of the inspected group of streets belongs to $C_{1}$, with the noisiest acoustic footprint. Finally, cluster $C_{3}$ describes a similar effect as the previous case of groups deaccelerating zones.

After, a further visual inspection of the studied street group was carried out. The area assigned to $C_{2}$ certainly showed signs of recent repaving, and zones assigned to$\;C_{1}$ correspond to the old pavement.

A straightforward interpretation of the cluster correspondence of a road-surface condition is possible if the road surfaces along each route have similar materiality at the time of installation. This condition is assumed for the roads considered separately for each case study. From this point of view, the cluster with higher amplitude of acoustic footprint can be linked to the old areas, while new areas would belong to a lower amplitude cluster. Details of the visual inspection and the comparison with cluster estimation are presented at the end of the section in Table 3.

A comparison is made between interpretation of the resulting clusters and visual inspection. The term “estimation” is used since there are no previously assigned class labels to compare the detections. Nonetheless, it was possible to obtain the length of the road sections of each circuit $l_{RS}$, which were visually labeled as “renewed” or “distressed”. Then, a ratio between $l_{RS}$ and the length assigned to each cluster $l_{C}$ is calculated. The length assigned to each cluster $l_{C}$ was obtained by counting the total length of segments assigned to each cluster in the geoprocessing step. In the same way, the total length of the “no-assigned” sections $l_{na}$ and the total length of “no-data” sections $l_{nd}$ were counted. Each section of the route has a dominant cluster $C_{k}$, which covers the greatest length of that section. Using this cluster, the condition of each section was rated.

The dominant clusters on each route section agree with the discriminated wheel-path condition obtained from the cluster interpretation and with the assigned condition by means of visual inspection. Then, the correct identification of each route section is estimated by the ratio between the dominant cluster length $l_{C}$ and the total distance of the route section, excluding the part that has not been considered on the datasets $l_{RS}-l_{nd}$, in percentage. The percentage of the route that was not clearly identified and the percentage of the route that did not contribute data to the study are also presented, using $l_{na}/l_{RS}$ and $l_{nd}/l_{RS}$, respectively.

The results show that the surface discrimination for two pavement condition categories was obtained from the clusters within the reduced feature space. In the roadway of the University campus, the detection of both renewed and distressed sections shows more separable resulting clusters in the feature space (e.g., ΔLeq = 7.2 dB), and the a priori knowledge of the superficial roadway status allowed a high estimation of the real condition of the road. In particular, the renewed section length was detected up to 96% and the distressed section length up to 76%.

On the other hand, the system is less effective in road environments with greater variability. These are the cases of the urban avenue and the urban circuit, where the presence of a third cluster is related with external urban factors instead rolling noise. Therefore, this cluster was not considered for the estimation of the road surface condition. In addition, the classes proposed by the number of clusters are closer to each other into the feature space (i.e., ΔLeq < 3 dB). As a result, the discrimination rate of distressed areas between 60% and 72% was achieved for urban roads environment.

## 4. Discussion

The results of the automatic discrimination of the asphalt surface condition over the wheel-path interaction zone have been compared with the conventional visual inspection technique, which is performed on reference points of the road. In addition, local road evaluations were generalized to make them comparable with the results of the large length inspection attained from the proposed method. As a result, the discrimination rate presented in Table 3 can be underestimated

For all urban-roads study case, the corresponding $C_{3}$ appears in localized deceleration zones. It suggests that several low-noise data observations do not contain information purely related to tire-road noise. The speed limits and the car used for this traffic environment would make it difficult to record signals without contamination, such as ambient noise or phenomena related to sound reflections. This effect would be present in all clusters due to data acquired at speeds very close to the crossover speed (35 km/h) but mostly assigned to $C_{3}$.

Due to the location of the microphones, BTT method depends on the wheel path, which is the contact area between the tire and the road during normal traffic. Therefore, the assigned group is not necessarily the same for all passes neither for both tires at the same time. However, with increasing passes, the detections show homogeneity across road segments, and the subsequent geoprocessing step allows smoothing the cluster assignment by a majority vote summary.

This acquiring system proposed allows to process signals from two microphone positions independently. This suggests that two cars with similar engine and tire characteristics and with only one instrumented tire, would provide similar results. However, the influence of driver and vehicle variability must be considered in the clustering interpretation. This can lead to collecting information collaboratively in the horizon.

- This method, based on the clustering of the acoustic features of rolling noise, provides an unsupervised alternative for the discrimination of the asphalt surface status along the trajectory followed by a vehicle wheel. Notwithstanding, certain possible improvements, such as the selection of the vehicle and the placement of the microphone for data acquisition, should be considered in future works.
- The placement of the microphone in the wheel housing produces signals that are not “purely” from the interaction between the tire and the road. In fact, there could be other types of sounds, both from the outside (when driving on busy roads) and from the vehicle itself (noise from the exhaust pipe or from the ventilation of the wheels themselves). The presence of these other sounds could negatively affect the classification process by increasing the background noise and masking the signal containing the contact surface information. However, most of these components are not sensitive to the type of road surface the vehicle is driving on. Therefore, the filtering, feature extraction, and dimensionality reduction processes have allowed to minimize their influence, as their acoustic fingerprints are separable. These non-overlapping classes were also observed in experiments with supervised classifiers with similar microphone placement [5,25,32,58]. Furthermore, the impact of these spurious sound phenomena is minimised by processing multiple observations at the same location using the geoprocessing step.
- The selection of the speed of reference in the linear model could be improved in future works. Although the literature reports the linear relationship of the TPIN levels in dB and $log\frac{speed\;\left[\mathrm{km}/h\right]}{speed_{ref}\;\left[\mathrm{km}/h\right]}$ over a wide range of speeds, the speed of reference was considered constant throughout the experiments for homogeneous data processing. The analysis could be improved by applying different values of the speed reference based on standard recommendations as is proposed by ISO [46] and CNOSSOS-EU for traffic noise emissions modelling.
- The asphalt discrimination rate in urban scenarios could be improved with the application of this acoustics-based method using electric vehicles, due to the lower crossing speed (<35 km/h) as reported in [22].
- The detection of road surface quality by unsupervised learning has been evaluated by comparisons with applications of supervised classification metrics (i.e., accuracy) [35]. On the other hand, the present work proposes the “Estimation of road- section discrimination” which is based on the actual length of the road.
- Comparison of the signals acquired by two or more microphones (e.g., one for each tire) could be included in future research steps. This would improve the detection of wear of the pavement, such as potholes, cracks, and bumps. A shorter-time window could also be included for impulsive noise events processing.

## 5. Conclusions

The main contribution of this paper is to present the performance of asphalt surface characterization based on the TPIN signal produced along the wheel path. This unsupervised technique allows the automatic discrimination of a small number of clusters related to predominant asphalt superficial condition.

Moreover, this method takes advantage of some improvement opportunities detected in previous related works, such as the inclusion of driving condition influences and the detection of the differences over the asphalt length without a catalogue of damages.

Three kinds of scenarios were considered to check the performance of the methodology such as a reference route, one urban avenue, and one urban street circuit. All these pavements possessed same characteristics of regular aggregate compositions, i.e., no one of them were neither open-graded pavements, rubber asphalts or poroelastic surfaces. These measurements were limited to weather conditions without rain and dry asphalt. The conclusions of the study are as follows:

- The superficial condition of the studied roads is closely related to the rolling sound footprint and TPIN amplitudes in the frequency and time domains. These relations allow the interpretation of the clustering results.
- An advantage of the application of UL over supervised techniques is the possibility of detecting areas with homogeneous rolling noise footprint without knowledge of the current road status. These localized zones are related to the homogeneous condition of the road status (deteriorated or not). The results were compared throughout further conventional visual inspections.
- The implemented methodology has allowed the automatic and continuous discrimination of the state of the asphalt surface along the wheel trajectory. From these results, the surface discrimination of the wheel path on single lane roads can reach 92 % (i.e., the reference road and the urban street circuit). Multiple observations allow to evaluate better the TPIN from a narrower wheel track area.
- Whereas in the case of the urban scenarios of roads with more than one lane, the discrimination rate decreases up to 57%. This because of the discrimination system must deal with different variables such as the speed limit, traffic flow, and a wider inspected area. Especially, when the vehicle changes lanes during each trip as it could happened during naturalistic driving behaviour.
- The present acoustic-based method allows the inspection of road facilities with nonstop traffic inspections, non-destructive approach, and opportunistic scenario.
- The mapping report contributes to pavement management through visual information. The surveyed areas producing different TPIN footprints assist in road maintenance planning, traffic noise mitigation activities, road condition warning reports.
- In the present research phase, only corrections due to driving characteristics (speed and acceleration) were included. Future developments are also expected to incorporate corrections due to the variability of other conditions during driving, such as vehicle load, driver behaviour, tire inflation pressure, tire tread pattern, temperature, humidity, vehicle engine, pavement materiality, etc.
- The technique could be improved for the detection of punctual defects such as potholes or manholes through refined time windowing in the signal processing and spatial resolution in the geoprocessing.
- However, the consideration of these new conditions will surely imply complexity in the clustering interpretation.

## Figures and Tables

**Table 2:** Elements of dataset and the length of the inspected routes.

Roadway ID	Circuit Length [km]	Passes	Travel Length Approx. [km]	Dataset Elements [n] 1-s Readings (Speed ≥ 35 km/h)
Data for lineal model			39.00	4504
Reference road	2.74	4	10.96	1430
Urban avenue	5.82	4	21.52	2266
Urban street circuit	2.81	4	11.24	1998

**Table 3:** Discrimination rate of the surface of wheel-path interaction zone by clustering.

Roadway ID	Circuit Length [km]	Road-Section by Visual Inspection. “Actual Condition”	Road-Section Length $l_{RS}$ [km]	Clustered Sections Lengths after Geoprocessing $l_{C}$ [km]			“Not Assigned” Section [km]	“No Data” Section [km]	Dominant Cluster $C_{k}$ on Road Sections	Estimation of the Road-Section Discrimination [%]	Percentage of Route without Clear Discrimination [%]	Percentage of No-Processed Route [%]
				C1	C2	C3	$l_{na}$	$l_{nd}$		$\frac{l_{C}}{l_{RS}-l_{nd}}*100$	$\frac{l_{na}}{l_{RS}}*100$	$\frac{l_{nd}}{l_{RS}}*100$
University campus	2.54	renewed	1.54	**1.42**	0.00	-	0.00	0.12	**C1**	92.21	0.00	7.79
		distressed	1.00	0.16	**0.76**	-	0.00	0.08	**C2**	76.00	0.00	8.00
Urban avenue	5.39	renewed	1.64	0.18	**0.94**	0.22	0.20	0.10	**C2**	57.32	12.20	6.10
		distressed	3.74	**2.27**	0.46	0.52	0.30	0.20	**C1**	60.53	8.02	5.33
Urban street circuit	2.81	renewed	0.48	0.04	**0.36**	0.06	0.02	0.00	**C2**	75.00	4.17	0.00
		distressed	2.35	**1.70**	0.34	0.18	0.07	0.06	**C1**	72.34	2.98	2.55

## Appendix A

**Table A1:** Features based on the triangular filters and the coefficients applied to the linear correction of the influence of speed and acceleration on TPIN levels.

Filter	Triangular Filter $T_{f}$			Coefficients		*p*-Values		*R* ^2^	Filter	Triangular Filter $T_{f}$			Coefficients		*p*-Values		*R* ^2^
	$f_{L}$	$f_{c}$	$f_{U}$	Bs Speed	Ba Accel.	Speed	Accel.			$f_{L}$	$f_{c}$	$f_{U}$	Bs Speed	Ba Accel.	Speed	Accel.	
**1**	50.0	65.0	80.0	38.7	2.2	0.0	0.0	76.7	**26**	553.9	593.3	635.5	33.3	1.7	0.0	0.0	87.7
**2**	65.0	80.0	95.0	37.4	1.8	0.0	0.0	78.9	**27**	593.3	635.5	680.8	33.6	1.7	0.0	0.0	86.7
**3**	80.0	95.0	110.0	32.9	1.6	0.0	0.0	74.3	**28**	635.5	680.8	729.2	32.0	1.9	0.0	0.0	85.3
**4**	95.0	110.0	125.0	25.2	1.1	0.0	0.0	60.5	**29**	680.8	729.2	781.1	33.6	1.8	0.0	0.0	85.6
**5**	110.0	125.0	140.0	22.2	0.9	0.0	0.0	57.8	**30**	729.2	781.1	836.7	37.3	1.7	0.0	0.0	87.5
**6**	125.0	140.0	155.0	22.5	0.9	0.0	0.0	59.7	**31**	781.1	836.7	896.2	36.6	1.8	0.0	0.0	86.5
**7**	140.0	155.0	170.0	23.5	0.9	0.0	0.0	60.8	**32**	836.7	896.2	960.0	36.1	1.7	0.0	0.0	87.0
**8**	155.0	170.0	185.0	25.6	0.9	0.0	0.0	65.5	**33**	896.2	960.0	1028.4	34.7	1.7	0.0	0.0	85.8
**9**	170.0	185.0	200.0	27.2	0.8	0.0	0.0	65.0	**34**	960.0	1028.4	1101.5	35.5	1.7	0.0	0.0	84.5
**10**	185.0	200.0	215.0	27.2	0.9	0.0	0.0	67.7	**35**	1028.4	1101.5	1179.9	36.0	1.8	0.0	0.0	81.5
**11**	200.0	215.0	230.0	25.6	0.8	0.0	0.0	65.8	**36**	1101.5	1179.9	1263.9	35.0	1.6	0.0	0.0	79.2
**12**	215.0	230.0	245.0	20.5	1.2	0.0	0.0	57.0	**37**	1179.9	1263.9	1353.9	34.3	1.8	0.0	0.0	79.2
**13**	230.0	245.0	260.0	18.0	1.1	0.0	0.0	53.1	**38**	1263.9	1353.9	1450.2	34.4	1.8	0.0	0.0	82.5
**14**	245.0	260.0	278.5	16.9	1.2	0.0	0.0	48.7	**39**	1353.9	1450.2	1553.4	33.8	1.7	0.0	0.0	81.5
**15**	260.0	278.5	298.3	17.7	1.5	0.0	0.0	51.2	**40**	1450.2	1553.4	1664.0	31.9	1.6	0.0	0.0	80.9
**16**	278.5	298.3	319.6	20.1	1.6	0.0	0.0	61.2	**41**	1553.4	1664.0	1782.4	32.0	1.7	0.0	0.0	82.2
**17**	298.3	319.6	342.3	25.0	1.6	0.0	0.0	71.8	**42**	1664.0	1782.4	1909.3	28.4	1.9	0.0	0.0	78.8
**18**	319.6	342.3	366.7	30.4	1.8	0.0	0.0	72.9	**43**	1782.4	1909.3	2045.2	31.4	2.0	0.0	0.0	79.7
**19**	342.3	366.7	392.8	29.5	1.7	0.0	0.0	73.7	**44**	1909.3	2045.2	2190.7	27.7	1.9	0.0	0.0	78.4
**20**	366.7	392.8	420.7	30.4	1.9	0.0	0.0	83.4	**45**	2045.2	2190.7	2346.6	27.5	2.1	0.0	0.0	77.0
**21**	392.8	420.7	450.7	30.9	2.0	0.0	0.0	84.1	**46**	2190.7	2346.6	2513.6	27.6	2.0	0.0	0.0	82.2
**22**	420.7	450.7	482.7	31.6	1.8	0.0	0.0	84.3	**47**	2346.6	2513.6	2692.5	27.6	1.8	0.0	0.0	83.0
**23**	450.7	482.7	517.1	33.6	1.8	0.0	0.0	84.2	**48**	2513.6	2692.5	2884.2	28.1	1.9	0.0	0.0	81.3
**24**	482.7	517.1	553.9	33.2	1.6	0.0	0.0	86.5	**49**	2692.5	2884.2	3089.4	27.9	1.9	0.0	0.0	79.6
**25**	517.1	553.9	593.3	32.7	1.7	0.0	0.0	87.5	**50**	2884.2	3089.4	3309.3	28.1	1.9	0.0	0.0	79.8

In, $f_{L}$, $f_{c}$, and $f_{U}$ are the lower, central, and upper frequencies of the triangular filters, respectively. The coefficients for Equation (1) by each triangular filter are Bs for the speed and Ba for the acceleration. Additionally, the *p*-values and the coefficient of determination $R^{2}\left(\%\right)$ are presented. The highlighted filters (20 to 50) correspond to the selected 31 frequency features.
